# Editorial: Reducing animal use in carcinogenicity testing

**DOI:** 10.3389/ftox.2024.1538905

**Published:** 2024-12-18

**Authors:** Jan Willem Van der Laan, Joseph Manuppello

**Affiliations:** ^1^ Retired from Medicines Evaluation Board, Utrecht, Netherlands; ^2^ Research and Regulatory Affairs Department, Physicians Committee for Responsible Medicine, Washington, DC, United States

**Keywords:** regulatory toxicology, carcinogenicity testing, 3Rs, Non-genotoxic risk assessment, alternative methods

## Introduction

Internationally, new pharmaceuticals for human use are among the chemical substances for which regulatory authorities require the evaluation of carcinogenic potential with long-term studies in rats and mice. Large numbers of animals are used in these studies and in many cases, they endure prolonged distress. Therefore, reducing the number of animals used, as well as the duration of exposure, benefits animals. Reducing the time needed for preclinical development also benefits patients.

Although focusing mainly on studies in rats, the Addendum to the ICH S1B (R1) (International Council for Harmonisation, 2022) Guideline on testing for carcinogenicity of pharmaceuticals promises to decrease the number of long-term studies in both rats and mice. The greatest reduction in animal use will be achieved by assessing the Weight of Evidence (WoE) to determine whether a study in rats is likely to add value. Consistent with the original S1B Guideline, the Addendum also prioritizes the use of carcinogenicity studies in transgenic mice, which reduce animal use and exposure compared to studies in wild-type mice.

## ICH-S1B related articles

Over 12 years, Bourcier et al. from the ICH Expert Working Group evaluated a dataset of 45 compounds for which a prediction of the outcome of the rat study was being tested. Bourcier et al. From industry partners in this process, Vahle et al. presents an in-depth discussion of the types of information sources that are available for the various factors in the WoE approach described in the Addendum. Bassan et al. contribute a similar commentary describing in detail the various steps in this WoE approach. Importantly, these authors emphasize that carefully planning the carcinogenicity evaluation process should start early in drug development.

## Relevant approaches from other fields

Using TempO-Seq and microarray data, Ledbetter et al. including authors with the US Environmental Protection Agency) report the development of a 5-day rat study that identifies gene expression biomarkers linked to tumorigenic activation by liver carcinogens. While this approach uses animals, it has the potential to reduce animal use and exposure compared to carcinogenicity studies. Further, it could be combined with general toxicity studies to support the WoE assessment to determine whether a carcinogenicity study in rats adds value, as recommended in the Addendum, without increasing the overall number of animals used. Hopefully, it will also facilitate the development of human-based *in vitro* transcriptomic methods.

From the field of agrochemical safety assessment, Goetz et al. highlight a similar move away from the rodent cancer bioassay. As the pharmacological target is not defined in this group of chemicals, defining the biological target is more difficult than with pharmaceuticals. The article addresses this difficulty using case studies that include read-across approaches.

## Specific cases

Both Keller et al. and Pillo et al. focus on specific compounds, the human pharmaceutical pregabalin (an antiepileptic also used as a mood-stabilizer) and the plasticizer bis (2-ethylhexyl) phthalate (DEHP), respectively. Intersecting with Ledbetter et al., Keller et al. describe *in silico* approaches in carcinogenic hazard assessment that emphasize toxicological modes-of-action that include oxidative stress, chronic inflammation, and cell proliferation.

Pillo et al. provide an overview of the carcinogenic activity and molecular mechanisms of DEHP, identifying multiple molecular signals that appear to be involved in its carcinogenicity. While some endpoints, such as PPARα-activation, are probably not relevant to human risk assessment, other mechanisms might also be involved. DEHP did not induce transformation in BALB/c-3T3 cells; however, the transcriptomic results demonstrate specific modulations of genes and cell-regulation signaling pathways. Such “transformics” assays show promise for minimizing the use of animal testing for carcinogenicity assessment.

## Future use of databases

Finally, Karamertzanis et al. describe a database based upon the use of the pharmacotherapeutic criteria (ATC-code) and species/strain information in 520 carcinogenicity studies. As the full database also includes information from repeat-dose toxicity studies, it can be used to correlate histopathological findings with carcinogenicity, providing support for using WoE assessments to determine whether carcinogenicity studies are likely to add value.

## Discussion and conclusion

These eight papers clearly fit into an important development in the toxicological world, i.e., the reduction of animal use in risk assessment. In addition to human pharmaceuticals, these contributions describe important approaches for agrochemicals and can be applied in other fields, such as industrial chemicals.

The Addendum to the ICH S1B (R1) International Council for Harmonisation (2022) indicates that “emerging technologies” might be used for additional investigations. The contributions to this Research Topic, such as Ledbetter et al., Goetz et al. and Keller et al., all illustrate the value of these emerging technologies.

More than 20 years ago, the use of transgenic mice was introduced as an additional option with various pro-oncogenic approaches, e.g., by introducing human ras-oncogene in rasH2-Tg mice. At that time, it was an emerging technology to enhance the detection of human relevant nongenotoxic compounds based upon a mechanistic principle. The original ICH S1B Guideline clearly indicates its usefulness as an alternative to life-time studies with wild type mice.

In this Research Topic, none of the papers on new methodologies refer to the use of rasH2-Tg mice, although the carcinogenic potentials of various compounds in the Prospective Evaluation Study reviewed by Bourcier et al. were determined based partially on a study with this strain. The question can be raised whether the added value of the use of rasH2-Tg mice can still be recognized.

The data from the Prospective Evaluation Study reviewed by Bourcier et al. indicate that by applying the WoE approach, even without data on recent emerging technologies, 27% of the studies could have been dismissed (unanimous decisions in 12/45 CAD’s in Cat. 3A/3B), which is already an important result. The emerging technologies described in the other contributions to this Research Topic raise hope that the percentage of WoE assessments indicating there is “no-added value” in conducting a study in rats will increase in the near future.
